# Identifying safe care processes when GPs work in or alongside emergency departments: a realist evaluation

**DOI:** 10.3399/BJGP.2021.0090

**Published:** 2021-10-19

**Authors:** Alison Cooper, Andrew Carson-Stevens, Michelle Edwards, Freya Davies, Liam J Donaldson, Pippa Anderson, Matthew Cooke, Jeremy Dale, Bridie Angela Evans, Barbara Harrington, Julie Hepburn, Peter Hibbert, Thomas Hughes, Alison Porter, Aloysius Niroshan Siriwardena, Helen Snooks, Adrian Edwards

**Affiliations:** Division of Population Medicine, School of Medicine, Cardiff University, Cardiff, UK.; Division of Population Medicine, School of Medicine, Cardiff University, Cardiff, UK.; Division of Population Medicine, School of Medicine, Cardiff University, Cardiff, UK.; Division of Population Medicine, School of Medicine, Cardiff University, Cardiff, UK.; London School of Hygiene and Tropical Medicine, London, UK.; Swansea Centre for Health Economics, Swansea University.; Division of Health Sciences, Warwick Medical School, Coventry, UK.; Division of Health Sciences, Warwick Medical School, Coventry, UK.; Institute of Life Science 2, Swansea University Medical School, Swansea, UK.; PRIME Centre Wales.; PRIME Centre Wales.; Australian Institute of Health Innovation, Macquarie University, Australia.; John Radcliffe Hospital, Oxford, UK.; Institute of Life Science 2, Swansea University Medical School, Swansea, UK.; School of Health and Social Care, University of Lincoln, Lincoln, UK.; Institute of Life Science 2, Swansea University Medical School, Swansea, UK.; Division of Population Medicine, School of Medicine, Cardiff University, Cardiff, UK.

**Keywords:** clinical decision making, emergency departments, general practitioners, human factors, patient safety

## Abstract

**Background:**

Increasing pressure on emergency services has led to the development of different models of care delivery including GPs working in or alongside emergency departments (EDs), but with a lack of evidence for patient safety outcomes.

**Aim:**

This study aimed to explore how care processes work and how patient safety incidents associated with GPs working in ED settings may be mitigated.

**Design and setting:**

Realist methodology with a purposive sample of 13 EDs in England and Wales with different GP service models. The study sought to understand the relationship between contexts, mechanisms, and outcomes to develop theories about how and why patient safety incidents may occur, and how safe care was perceived to be delivered.

**Method:**

Qualitative data were collected (observations, semi-structured audio-recorded staff interviews, and local patient safety incident reports). Data were coded using ‘if, then, because’ statements to refine initial theories developed from an earlier rapid realist literature review and analysis of a sample of national patient safety incident reports.

**Results:**

The authors developed a programme theory to describe how safe patient care was perceived to be delivered in these service models, including: an experienced streaming nurse using local guidance and early warning scores; support for GPs’ clinical decision making, with clear governance processes relevant to the intended role (traditional GP approach or emergency medicine approach); and strong clinical leadership to promote teamwork and improve communication between services.

**Conclusion:**

The findings of this study can be used as a focus for more in-depth human factors investigations to optimise work conditions in this complex care delivery setting.

## INTRODUCTION

Patient safety is described by the World Health Organization as a serious global public health concern,^1^ but new healthcare service models are frequently introduced without evidence for patient safety outcomes.^2^ An example is the implementation of GP services in or alongside EDs, advocated (and resourced) in England as an approach to manage increasing patient demand.^3^ As a result, these service models have increased in England from 81% to 95% (2017–2019),^4^ despite a lack of evidence for their effectiveness and safety outcomes.^5^

Urgent and emergency healthcare services are complex adaptive sociotechnical systems.^6^ The environment is unpredictable and challenging, with pressures of time and uncertainty, as a wide variety of patients present with undifferentiated problems.^7^ GP service models associated with EDs may be situated inside the ED, integrated with the emergency medicine service (inside– integrated) or as a separate parallel service (inside–parallel); or outside the ED, on the hospital site (outside–onsite) or separate from the hospital site (outside–offsite).^7^ Previous analysis of a sample of national patient safety incident reports describing diagnostic error associated with these service models (UK Coroners’ and National Reporting and Learning System reports) highlighted key areas for improvement, including: streaming processes; GPs’ clinical decision making; and communication between services.^8^ Understanding how work conditions may influence the way GPs work (human factors) and how processes can be optimised to mitigate such events and support GPs in these different service models is overdue.

Quantitative analysis of routinely collected hospital data may not capture the complexity of these services, how they work, and why outcomes may occur, and may also be limited by poor data quality.^9^ Qualitative methods are required to improve understanding about how complex nonlinear phenomena may contribute to patient safety incidents (a ‘Safety-I’ approach).^10^ They can also be used to explore how human factors enable work to be conducted safely in both expected and unexpected conditions, understanding work-as-done rather than work-as-intended (a ‘Safety-II’ approach).^11^ Theory-driven realist methods are well suited to evaluating such services to explain what works, for whom, in what circumstances, and why, incorporating formal theory to describe how contextual factors may facilitate or inhibit patient safety outcomes.^12^

**Table:** How this fits in

New healthcare service models are often introduced without evidence for patient safety outcomes; this includes GPs working in or alongside emergency departments (EDs). In the present study, realist methodology was used to explore what works, for whom, in what circumstances, and how, in a purposive sample of 13 EDs, to understand how and why patient safety incidents may occur, and how safe care was considered to be delivered. Working in ED settings may influence GPs’ clinical decision making: working with a usual GP approach; a more cautious GP approach; choosing for different patients whether to maintain a GP approach or adopt an emergency medicine approach; or adopting an emergency medicine approach. Experienced streaming nurses, clear governance processes to support the intended GP role, and strong clinical leadership to encourage communication and teamwork between services were perceived to facilitate safe patient care in these complex care delivery settings.

This study aimed to test and refine initial theories developed through an earlier rapid realist review,^13^ and analysis of national patient safety incident reports,^8^ with qualitative data from a purposive sample of 13 case study sites, to explain how care processes are most likely to prevent or mitigate patient safety incidents associated with GPs working in ED settings.

## METHOD

Realist methodology is a theory-driven approach to evaluation, identifying mechanisms that explain how or why contexts relate to outcomes to generate theories described as context–mechanism– outcome (CMO) configurations; specific terminology is defined in Box 1.^12^ This study followed RAMESES reporting and publication standards (see Supplementary Table S1).^14^

**Box 1.** Realist definitions

Context	Pre-existing conditions that influence the success or failure of different interventions or programmes
Mechanism	Characteristics of the intervention and people’s reaction to it; how it influences their reasoning
Outcome	Intended and unintended results of the intervention because of a mechanism operating within a context
Initial rough theory	An early theory, informed by available evidence, about how, why, for whom, and in what circumstances the intervention is thought to work, described as a context-mechanism-outcome configuration
Refined theory	An initial theory that has been refined using primary or secondary evidence
Programme theory	An overall high-level theory summarising how the intervention works, developed using the theories refined from the data
Formal theory	Existing social theories used as a lens through which to examine the data; otherwise known as middle-range or substantive theory

### Case site selection

Case sites (hospitals) were recruited from responders to a national survey, followed up by a key informant telephone interview with the site clinical lead.^15^ An online survey was sent to the clinical directors of all type 1 EDs (24-hour consultant-led units with full resuscitation facilities) in England (*n* = 171) and Wales (*n* = 13) on behalf of both the ‘GPs in EDs’^16^ and ‘General Practitioners and Emergency Departments’ (GPED)^17^ study teams (both with the same funder). The aim was to capture data about the GP services being provided in or alongside EDs and how they worked, to inform a taxonomy of GP–ED models for both studies. The published taxonomy contains further information about the survey process and results.^7^

This study had survey responses from 71 English and 6 Welsh sites (*n* = 77/184, 42%). The GPED team also provided data for 41 English departments from Care Quality Commission reports and NHS England, totalling information on 62% (*n* = 118/189) of type 1 EDs in England and Wales.^7^ As a gauge of non-response bias, the study’s 71 English survey responders included 82% (*n* = 58/71) who had applied for capital bid (GP streaming) funding, compared with 84% of the 100 non-responders in England. The 13 case sites were purposively selected according to variables listed in Box 2 to ensure they covered a range of models and contexts. The included sample of anonymised case study sites and characteristics is listed in Supplementary Table S2. Classified by the taxonomy, these included:

- three ‘inside–integrated’ models;
- four ‘inside–parallel’ models (one was reclassified following the visit);
- three ‘outside–onsite’ models; and
- three sites without a GP service model.

**Box 1.** Variables used to purposively sample emergency departments

GP service implemented in the emergency department since 2010 Different service models: inside–integrated; inside–parallel; outside–onsite; and sites with no GP service Spread of geographical locations in England and Wales Variety of contexts — including hospitals in rural and urban locations/towns, small and large hospitals, and higher versus lower attendances Variation in patient streaming methods — who streams, streaming criteria, and guidance Variation in the physical layout of the GP service in relation to the ED Variation in relationships with the GP out-of-hours service

### Data collection

Two researchers visited all sites with a GP service (*n* = 10) for 2–4 days (mean 3 days) and individually conducted a 1-day visit at control sites between January 2018 and April 2019. The study conducted observations, including informal interviews; semi-structured audio-recorded realist interviews; and analysed local patient safety incident reports.

#### Observations

The authors spent time in reception and clinical areas (but did not observe clinical consultations) and observed triage and streaming processes. The authors opportunistically introduced themselves to a wide range of staff and asked questions to test various theories. When it was not possible to talk with staff, the authors observed how the systems worked, taking handwritten fieldnotes that were typed the same evening.

The authors met every 2 hours during the day to discuss findings, refer to the list of initial theories, and identify evidence gaps for theory testing. Eight visits were conducted midweek (usually Monday to Wednesday), with six visits, including observations, in the evening. Two visits were conducted over a weekend. Where possible, an exit interview was held with the clinical director, before leaving, to assist theory refinement.

#### Staff realist interviews

The clinical director, ED staff, and GPs were recruited during case site visits for audio-recorded interviews on site in a private area, or, later, via telephone; these were then transcribed verbatim. The realist teacher–learner interview technique was used where initial theories are presented to the participant to explore how mechanisms in different contexts may result in intended and unintended outcomes; see Supplementary Table S3 for an example of the interview guide.^18^

#### Local patient safety incident reports

Up to four separate requests were made for reports relevant to the GP service at each participating site (excluding those with no GPs). These data were usually in the form of printed anonymised reports that were given, in person, to the researcher who copied the free text directly onto a remotely accessed secure computer platform (PISA platform) at Cardiff University.

### Data analysis

Themes were analysed based on the initial theories generated through a rapid realist review,^13^ and analysis of national patient safety incident reports (Table 1).^8^ NVivo (version 11) was used to support categorisation of data, with separate folders for documents relevant to each GP service model (inside–integrated, inside– parallel, outside–onsite, and no GPs). The authors coded data using ‘if, then, because' statements to capture the nuance of different contexts.^19^ The level of qualitative evidence was classified supporting these statements in a hierarchy based on meta-ethnography principles.^20^ Findings were discussed weekly within the study team and co-applicants, including patient and public representatives, going back to the data for further information or clarification as required.

**Table 1.** Initial and refined theories described as context–mechanism–outcome configurations

**Process**	**Initial theory from rapid realist review and national patient safety incident reports**	**Refined theory from case site qualitative data**	**Example of supporting verbatim quote**
1. Streaming decisions	If patients present to the emergency department (C) but the **streaming nurse is unclear** which patients are appropriate for the GP service (because of unclear guidance or inexperience) (M) or the **initial assessment is inadequate** (limited history or lack of basic physiological observations) (M) then **higher-risk patients may be streamed** to the GP service (O).	If there is **adequate staffing** to meet **standard triage targets** and streaming is conducted by an **experienced nurse**, using **early warning scores** , with **guidance based on the local GP service provision**, and there is **good communication between services** about capacity and skillset (C) then the nurse **understands which conditions are appropriate** for the GP service (M), uses **clinical judgement and early warning scores** to identify sick patients (M), is **aware of the flow and capacity** in different streams in the department (M), and **the department modifies the process based on experience** and learning (M) then **patients with appropriate conditions** will be streamed (O).	*‘In the end what it really boils down to is having an* ***experienced member of staff*** *, working within* ***fairly broad parameters*** *of what is appropriate and what is not … There’s always a temptation amongst the nursing staff to put a less experienced person on streaming or triage simply to keep the most skilled people seeing the sickest patients, but that’s definitely the wrong thing to do,* ***we have to have experience up front because it’s an extremely important job getting them in the right place I think*** *.’* (Emergency consultant, hospital 10, outside–onsite model)
2. GPs’ clinical decision making^a^	GPs working in or alongside emergency departments seeing streamed patients (C) may be **influenced by the prior decision making of the streaming nurse** and **at risk of framing or anchoring cognitive biases** (M), or may **incorporate their usual community pre-test probability of serious illness** into their diagnostic reasoning and **be at risk of availability or representativeness cognitive biases** (M), or may have **inadequate knowledge or skillset** for the patients’ condition (M) and may be at **risk of mismanaging** the patient (O).	If GPs work in emergency departments with **clear governance processes**; are **aware of their intended role** and expectation depending on their **experience, skillset, and patient demand** (C); use **communication skills** to gather patient information for hypothesis generation (M); **actively consider prevalence of more serious diseases** that may present to the emergency department setting (M); use **clinical skills to rule out serious diagnoses** (M); **refer to guidance when acute investigation/referral may be necessary** to exclude serious disease (M); and use **safety netting** to help manage diagnostic uncertainty (M), then **safe patient care will be facilitated** (O).	*‘I think that* ***the group of patients I see in A&E is very different*** *to the patients that I see in general practice, so my level of concern,* ***I’m quicker to be concerned with an A&E patient than I would be with a general practice patient*** *. The ability of the patients to self-select to come to A&E never ceases to amaze me …* ***Does it mean I investigate more? No*** *, I don’t think it does, it just means* ***I listen very carefully to the history and examine very carefully*** *. That’s my own perception.’* (GP, hospital 3, inside–integrated model)
3. Communication between services	If there is **poor communication** between the GP service and the emergency department service (C) because of a **lack of awareness about capacity** (M) and inadequate **referral pathways** (M) then patient assessment and treatment may be delayed (O).	Service models with **strong clinical leadership**, employing **experienced**, regular **GPs** with **opportunity for face-to-face communication** between services and **compatible computer systems** (C), with a **culture that encourages interprofessional communication and learning** (M) and **clinical leadership that promotes mutual respect** (M), **encourages communication** between services and teamwork to **facilitate safe patient care** (O).	*‘One of the* ***biggest things we didn’t expect*** *is the* ***effect of education****, that there’s a GP sitting in the department, seeing a frail elderly patient, the F2 is sitting next to them seeing a similar patient, and the* ***F2 is going “Why are you sending your patient home and I’m admitting mine?”*** *,* ***the amount of cross-fertilisation knowledge and support*** *was something that* ***we didn’t expect*** *that* ***we’ve really benefited from****.’* (Clinical director, hospital 14, inside–integrated model)

a *See Table 2 for further nuanced CMOs. C = context. M = mechanism. O = outcome.*

### Data synthesis

High-level themes and positive and negative outcomes, grouped with mechanisms at individual, department, and wider system levels, were used as a coding framework to categorise the statements across folders. The authors then used Microsoft Excel to consolidate statements into CMO configurations.^19^ The authors mapped CMO configurations developed for each GP service model between service models, synthesising using Pawson’s theory-building processes (juxtaposition, reconciliation, adjudication, and consolidation).^21^ The authors then developed a master Excel file to capture the whole process and populate the evidence (where available) for refined CMO configuration development.

### Incorporating formal theory

The authors then incorporated Croskerry’s dual-process model of reasoning to help explain GPs’ clinical decision making in ED settings.^12^^,^^22^ The model is based on two distinct decision-making processes: ‘System I’ and ‘System II’, originally described by Kahneman.^23^ ‘System I’ is fast, effortless, intuitive, and automatic; it is typical in diagnostic decision making by experienced clinicians who rely on pattern recognition or shortcuts (heuristics). ‘System II’ is slow, laborious, and logical.^23^ Croskerry applied this to clinical medicine and specifically to ED settings, describing the risks of cognitive biases in these settings.^22^^,^^24^^–^26 Findings were structured around the diagnostic process of generation, evaluation, and verification.^25^^,^^26^

### Stakeholder feedback

A national stakeholder event was held in Bristol, in December 2019, with a wide range of English- and Welsh-based attendees (*n* = 56), including policymakers and commissioners (*n* = 4), managers (*n* = 6), patient and public contributors (*n* = 13), ED doctors (*n* = 6), nurse practitioners (*n* = 2), GPs (*n* = 5), academics including study co-applicants (*n* = 17), and administrators (*n* = 3). Results were presented and feedback was collected from small-groupfacilitated discussions.

### Patient and public involvement

Patients and public members were involved in the study design and co-applicants in the funded study.^16^ They used their experience as NHS patients to contribute to this research. They supported recruitment and involvement of public and patient contributors to the stakeholder event. They were involved in discussing the draft data and preparing this article.^27^

## RESULTS

The authors included data from 66 staff interviews (Supplementary Table S4), fieldnotes from researcher observations at the purposive sample of 13 case sites, and 14 local patient safety incident reports relevant to the GP services (see Supplementary Table S5).

Clinical directors from nine of the 10 hospitals with a GP service had no patient safety concerns and did not describe any patient safety experiences related to the GP service. Two clinical directors from inside– integrated model sites perceived that, since GPs had been working in the department, overall patient safety had *improved* because more experienced, permanent GPs could also give advice to other staff members (hospitals 3 and 8). Safety incidents (and potential risks) regarding the GP service were described by senior staff at one case site with a GP service (inside–parallel model) and at a site that no longer had GPs working there. These supported the authors’ initial theories developed through earlier analysis of a sample of national patient safety incident reports describing diagnostic error associated with GP service models.^8^

Refined theories from these case site qualitative data focused on staff perceptions about how patient safety incidents, described in the authors’ initial theories, could be mitigated. They are presented under the following care processes: facilitating appropriate streaming decisions; supporting GPs’ clinical decision making; and improving communication between services (Table 1).

### Facilitating appropriate streaming decisions

Streaming nurses having difficulty identifying patients with appropriate conditions for the GP service was a common theme reported by ED doctors, nurses, and GPs across many case study sites (hospitals 4, 6, 9, and 10):

*‘It’s a bit hit and miss, it depends on what the help of the triage nurse is … sometimes patients you’re seeing are inappropriate, I’ve seen epiglottitis, which, really, I shouldn’t be seeing as a GP in A&E, but there’s lots of things that I could be seeing, which I don’t end up seeing, because they’re deemed to be an A&E case.’*

(GP, hospital 4, inside–parallel model)

An experienced advanced nurse practitioner described junior triage nurses’ inexperience as negatively influencing streaming decision making (hospital 10). He described how inexperienced nurses may not explore why patients had presented to the ED with the risk of missing ‘red flag’ symptoms, such as the possibility of cauda equina syndrome when a patient with chronic back pain presents with a history of incontinence. Understaffing in one case study site was also reported to delay the streaming process and triage because the streaming nurse also had to administer treatments. Many hospital case study sites were happy to share learning about how and why the streaming process worked well and how it had been modified, such as measuring basic observations, to ensure appropriate patients were streamed to the GPs:

*‘So, to give you an example of how we’ve learned … we had a child seen in the triage room, had the eyeball, went to Urgent Care … thankfully, the GP picked up that this was a sick child, got them to the resus room, ended up in intensive care. So, we had a very rapid learning and a very rapid PDSA [Plan Do Study Act] cycle there.’*

(Clinical director, hospital 3, inside–integrated model)

Guidance relevant to the local GP service was considered important but an experienced streaming nurse who could use their clinical judgement was felt to be essential (hospitals 7, 9, and 10). Appropriate communication between services allowed the streaming nurse to understand the capacity of the different streams (situation awareness), which again influenced streaming decisions (hospital 10).

### Supporting GPs’ clinical decision making

There was some evidence of GPs working within integrated service models seeing a wider range of patients, with some reports of fracture mismanagement (hospitals 4 and 14) and not following standard ED child safeguarding protocols (hospitals 3 and 4):

*‘A child was seen who was known to have had input from social services … and the GP had seen them, and they really should have rung social services just to alert them that the patient had been seen … but they just seemed to maybe not have quite the right level of concern and appreciation of the need to keep social services involved.’*

(Clinical director, hospital 4, inside–parallel model)

Unclear governance processes across different commissioning organisations, including job description, induction, and supervision requirements, were felt, by a senior consultant at one site, to contribute to confusion about which patients the GP service should be managing:

*‘I was concerned from the outset, really, about the lack of clarity behind where was the governance, what were they supposed to be seeing, was it within their normal scope of practice … We had an incident of a missed cervical spine fracture.’*

(Emergency consultant, hospital 4, inside–parallel model)

Four CMO configurations were developed from GP interview data (Table 2) to describe how working in ED settings influenced (or did not influence) their use of acute investigations and clinical decision making: a usual GP approach; a more cautious GP approach; the choice to take a GP approach or an emergency medicine approach; and the expectation to adopt an emergency medicine approach. Croskerry’s framework was then applied to consider the risks of cognitive errors at different stages of the diagnostic process and further refine these theories.^25^^,^^26^

**Table 2.** Factors described to influence different approaches to GPs’ clinical decision making in emergency department settings

**Impact on clinical decision making**	**Contextual factors**	**Mechanisms**	**Outcomes**	**Example of supporting verbatim quote**
Usual GP approach (perceived similar pre-test probability of serious disease)	If GPs who are **experienced** and **confident in their clinical skills** work in or alongside emergency departments without access to acute investigations, seeing patients **identified as being appropriate** for the GP service who **they perceive are a similar cohort to usual primary care** …	… they use their **clinical skills to ‘rule out worst case’**; are **comfortable with uncertainty**; use **‘safety netting’** techniques; and **admit patients** if they require acute investigation …	… to **safely use a GP approach**.	*‘The general theme we say to our GPs is* ***we shouldn’t work any differently here than we would do if we sat in our practices*** *, just because we’re in a hospital, we* ***don’t do anything differently****. If we need to admit people, we would obviously send them round as we normally would, in a practice, we’re not trying to be a mini A&E here and do anything differently.’* (GP, hospital 10, outside–onsite model)
More cautious GP approach (perceived higher pre-test probability of serious disease)	If GPs who are **experienced and confident in their** clinical skills work in or alongside emergency departments **with or without access** to acute investigations, seeing a **cohort of patients who they perceive to be higher risk** …	… **they incorporate this higher risk** into their clinical decision making; **consultations may be longer** for more thorough history taking; the **threshold for admission** or using other acute services for investigation **may be lower** …	… but many patients can still be **safely managed** by using a **GP approach**.	*‘If you* ***select the right patients to see****, as a GP in the department,* ***you should be able to deal with them in a similar way to you do in primary care*** *, but always just* ***having that slight radar on to think OK, is there something else going on*** *, do we need to do that little bit more?’* (GP, hospital 14, inside–integrated model)
Choose when to use a GP approach or an emergency department approach	If GPs with **additional emergency medicine skills and experience** work in emergency departments, seeing a **wider range of patients** with **access to acute investigations** …	… they can use their **clinical skills** to **assess risk** …	… to **choose** which patients can be safely managed by a usual **GP approach**, and which patients require acute investigation that they **can manage** using an **emergency medicine approach**.	*‘I tend to* ***categorise patients into three groups****: ones that I can* ***see and treat*** *and move on; ones that I* ***see and need to admit*** *or whatever I decide to do investigation wise, the decision is made early on; and then there’s the group in the middle where you’re uncertain whether this patient needs urgent admission or not, and you* ***use the investigations as a tool to help in that decision making*** *.’* (GP, hospital 3, inside–integrated model)
Expectation to adopt an emergency department clinician approach	If GPs work in emergency departments with **access to investigations**, where there is an **expectation to follow emergency department protocols**, or **governance responsibility is unclear,** or patients may have **already had investigations requested at triage** …	… GPs may feel that they are **expected to use these investigations** in the emergency department setting, or they may **become less confident** in their clinical skills, or have **medicolegal concerns** …	… and **may use investigations** for patients that they **would not have requested** if they had seen the patient in **usual primary care**.	*‘Thinking about defence,* ***if you don’t do tests when they’re right next to you*,** *and something were to happen, an adverse event,* ***you would have to be able to stand up to that and defend yourself*** *and* ***say why you didn’t do those tests****, so it’s tricky, and* ***I go through that all the time in my head****’* (GP, hospital 14, inside–integrated model)

#### Diagnosis generation

Generation of one or more diagnostic hypotheses begins early in the process, even before the clinical encounter with the patient has begun.^25^ GPs described making early clinical decisions, before the patient had been seen, based on the written triage notes, sending inappropriate patients back to the ED if necessary. Establishing the acuity of the patient’s condition was a common strategy described by GPs working in ED settings: categorising patients into those who required immediate medical attention or investigation and those who did not, rather than focusing on a specific diagnosis:

*‘It’s a different approach to working in the community where there’s usually nothing serious — it’s important not to miss a serious diagnosis. My approach: are there red flags? If not, can I treat it? Can I redirect?’*

(Comments from GP fieldnotes, hospital 9, inside–parallel model)

GPs’ perceptions of the ‘pre-test’ prevalence of serious disease, and whether the cohort of patients was similar to usual primary care patients or a higher-risk group, was described to impact their clinical decision making. GPs who perceived the cohort of patients as higher risk described a different level of concern and management of risk in the ED than in usual primary care. Initial information gathering from the patient, to understand why they had presented to the ED that day, and the background of the presenting complaint, was described by some experienced GPs as key to diagnostic decision making.

#### Diagnostic evaluation

Many GPs described excluding serious disease by ruling out the ‘worst case’ as the priority,^28^ often through careful history and examination, even if acute investigations were available. However, some GPs described a lower threshold to admit patients for investigation to exclude serious disease than they would in the community setting because of the increased prevalence of serious illness in ED settings.

#### Diagnostic verification

GPs described the priority being to exclude serious disease rather than making an actual diagnosis, which may not be possible because of limitations of the service:

*‘For me, my sort of mental triage system is “Do I need to admit you, yes or no, and can I deal with your issue now”, i.e. is it long term, in which case I probably can’t do very much, because I don’t have access to all of your notes and it’s not very practical, I can’t organise blood tests, I can’t organise scans … in which case I’ll have to send you back to your GP.’*

(GP, hospital 7, inside–parallel model)

The strategy of ‘safety netting’ was described as good practice to help manage diagnostic uncertainty — advising patients of potential worsening symptoms and when further medical advice should be sought.^29^

### Improving communication between services

Some hospital case sites were observed or reported to have limited communication between the GP and ED services. Incompatible computer systems were linked to two patient safety incidents, where patient assessment and treatment had been delayed (hospitals 3 and 6) (Supplementary Table S5). Receptionists at another case site described how they had three different computer systems to operate (for the ED, the GP–ED service, and the GP out-of-hours service), which led to duplicate patient entries on different systems and increased the likelihood of patients becoming lost in or between the different systems (hospital 7).

The layout of the department, with distance between the services limiting face-to-face communication, was felt to contribute to very limited communication between services at one site (hospital 11):

*‘We’re not very integrated with the ED and we don’t, we don’t feel very integrated, it still feels a bit us and them.’*

(GP, hospital 11, outside–onsite model)

An ‘us and them’ culture was observed and reported at another site (despite there being good opportunity for face-to-face communication with the GPs working out of an ED cubicle). At this site, juniors were not encouraged to ask the GPs for advice (hospital 4).

Another site, however, with a separate GP service, reported good communication through the senior nursing team, reviewing on-the-day capacity and skillsets, and moving staff between services to meet patient demand. The integrated GP services reported good communication, which was perceived to promote interprofessional learning. At these sites the GPs were employed on a regular, rather than locum, basis and there were good opportunities for face-to-face communication. GPs were described, not only to give clinical advice, but also to provide advice on primary care referral pathways, which ED staff reported as helpful. The authors observed a sense of multidisciplinary respect, trust, and teamwork, with clear ED clinical leadership (hospitals 3, 8, and 14). Strong GP leadership was seen at several case study sites, and was also reported to improve communication between the services and perceived to improve patient safety (hospitals 3, 10, and 14).

### Programme theory

This study’s findings were summarised in a programme theory, conceptualising the complexity of patients and pathways, to describe factors perceived to facilitate GPs delivering safe patient care in or alongside EDs (Supplementary Figure S1).

## DISCUSSION

### Summary

A programme theory was developed from observations, incident reports, and in-depth realist interviews to describe how safe patient care was perceived to be delivered when GPs work in or alongside EDs: experienced streaming nurses using early warning scores and local guidance to facilitate appropriate streaming decisions; clear governance processes to support GPs’ clinical decision making depending on the intended role (traditional GP or emergency medicine clinician); and compatible computer systems, experienced regular GPs, and strong clinical leadership to encourage communication and teamwork between the emergency and GP services.

### Strengths and limitations

Thirteen case study sites were purposively recruited for theory testing and refinement (including different service models in different sized hospitals, geographically spread across England and Wales). These were visited by the same two researchers who applied a consistent realist approach, testing and refining initial theories developed from the literature,^13^ and analysis of national patient safety incident reports,^8^ through realist teacher–learner interview techniques to explore how human factors influenced clinical risk and work-as-done rather than work-as-intended, when GPs worked in these settings.^11^^,^^18^ Longer visits and observations of clinical consultations, rather than interview data subject to staff and researcher perceptions, would have provided stronger evidence about ‘work-as-done’. Quantitative data are required to understand the effects of GPs working in EDs on safety outcomes, and on comparative effects with other professional groups.

The work was conducted as part of a larger study that dictated the sampling approach. Selecting sites from a national survey, with a response rate of 42%, limited sampling, although the authors had information on an additional 20% of hospitals, collectively with no evidence of non-response bias.^7^ No sites were recruited where GPs screened patients at the front door in a gatekeeper role; however, there may be departments operating this service model, of which the authors were unaware.

### Comparison with existing literature

GPs are recognised as low patient safety incident reporters, which may have contributed to the low number of local reports identified.^30^ There is little national guidance on which ED patients should be streamed to GP services,^5^^,^^31^ or by whom, with this study’s work supporting an experienced senior nurse over algorithmic methods.^32^

The dual-process model of reasoning has previously been applied to GPs working in a similar high-risk setting, out-of-hours, where they would not know their patients.^33^ Similar management approaches were described: dividing patients into those with serious (or potentially serious) conditions and patients likely to have non-serious conditions; and using ‘safety netting’ to manage diagnostic uncertainty.^33^ An initial patient-guided search, or the ‘golden minute’, is described as key in the information-gathering stage of the well-known Calgary–Cambridge clinical consultation.^34^^,^^35^ GPs described how they used their communication skills to gather information and to exclude serious disease, which may explain their reduced use of acute investigations.^36^

Communication failures, exacerbated by hierarchical differences and conflicting roles and role ambiguity, are associated with increased patient safety incidents,^37^^,^^38^ while interventions to improve communication between healthcare professionals, such as briefings or ‘huddles’, are associated with improved patient safety outcomes.^39^^,^^40^ Clinician involvement in leadership positions in hospitals is associated with improved quality of patient care.^41^

### Implications for research and practice

Since this work was conducted, urgent and emergency care services along with almost all NHS service provision have changed because of the COVID-19 pandemic, including telephone screening of ED ‘walk-in’ attendances^42^ and remote GP consultations.^43^ The learning from this work and human factors concepts can be applied when evaluating these new services for quality improvement purposes, including how streaming (or telephone screening and ‘care navigation’) decisions are made; how remote consultations may impact on GPs’ clinical decision making; and how to promote communication between new emergency service models to ensure improved patient safety.^44^

The complexity of the ED setting and the patients presenting to it, who are often seen by more than one staff member who do not know them, or their previous state of health, provides challenges for staff, including GPs. The authors propose a programme theory to describe how safe care is perceived to be facilitated when GPs work in or alongside EDs, including: appropriate streaming decisions; supporting GPs’ clinical decision making; and improving communication between services. These findings can be used as a focus for more in-depth human factors investigations to optimise work conditions in this complex care delivery setting.
